# Paradoxes in pediatric rehabilitation: building an interdisciplinary, total-child framework to promote effective interventions and life course well-being

**DOI:** 10.3389/fped.2025.1540479

**Published:** 2025-03-10

**Authors:** Sharon Landesman Ramey, Michael E. Msall, Craig T. Ramey

**Affiliations:** ^1^ Departments of Psychology and Pediatrics, Fralin Biomedical Research Institute at VTC, Virginia Tech, Roanoke, VA, United States; ^2^ Section of Developmental Pediatrics and Kennedy Research Center on Intellectual and Neurodevelopmental Disabilities, Department of Pediatrics, University of Chicago Comer Children’s Hospital, Chicago, IL, United States

**Keywords:** pediatric rehabilitation, early intervention, developmental disabilities, neuroplasticity, epigenetics, cerebral palsy, low birthweight, life course well-being

## Abstract

In this paper, we identify major paradoxes that have emerged from randomized controlled trials and longitudinal studies of diverse groups of young children with identified disabilities and risk conditions. We concentrate on the first three years of life because these coincide with a period of rapid changes in brain structure and function as well as dramatic expansion of a child's skills in motor, language, social-emotional, and cognitive domains. The paradoxes support a major revision in hypotheses about how effective interventions can alter a child's functioning and life course. The following conclusions derive from the paradoxes: (1) the intertwined biological and environmental influences on a child's well-being contribute more to functional outcomes than do the primary medical diagnoses and biological risks alone; (2) high-intensity, high-cost interventions that are well-timed, wholistic, and multi-domain can be more powerful and economical (i.e., yield higher “returns on investment”) than many treatments that initially appear less costly and easier to implement; (3) treatments that are individualized to the child and family, while adhering to evidence-backed treatment protocols, are among the most likely to result in large and long-lasting benefits compared to those that are solely individualized or adherent to a treatment protocol that does not make adjustments for the child; and 4) a clearly presented conceptual theoretical framework about human development can be a remarkably practical and informative tool in maximizing benefits of pediatric rehabilitation. We propose an interdisciplinary “total-child” platform – named the **I**nterdisciplinary **M**onitoring, **P**lanning, **a**nd **C**aring for the **T**otal-Child – **T**ogether (IMPACT2) Developmental Framework - to support forming strong partnerships to facilitate informed clinical and family decision-making as well as the design and conduct of scientific investigations. We encourage others to consider these paradoxes and the IMPACT2 framework to stimulate conversations and promote innovative family and community partnerships to realize greater impact from delivering effective pediatric rehabilitation interventions to all eligible children.

## Introduction

This paper has been germinating for a long time in the minds, research, and conversations of the three authors. Craig Ramey is among the first lifecourse developmental psychologists recruited through an NIH interdisciplinary program that admitted its first graduate students in 1966 and a pioneering scientist in infant learning research. He has conducted many randomized controlled trials (RCTs) testing the efficacy of early interventions developed to improve outcomes for vulnerable children and those with diagnosed developmental delays and disabilities. Michael Msall is a board-certified Neurodevelopmental and Behavioral Pediatrician who has conducted research and synthesized research findings in the areas of genetics, resiliency, low birthweight and prematurity, and developmental disabilities, including the development of innovative assessment measures of child functioning after neuroprotection interventions. Sharon Landesman Ramey is a developmental scientist with training in comparative and developmental psychobiology, ethology, and behavioral teratology who has conducted clinical trials since 1972 that test new interventions to improve learning and health outcomes in individuals with developmental disabilities (including children once deemed “untreatable”) and children with high environmental risks.

In 2022 at a national meeting, after a long, wide-ranging conversation about changes and opportunities in our fields, we pledged to continue sharing our thoughts and to write about some of the research findings that we think could transform the lives of vulnerable young children, but *have not yet been fully implemented.* These potentially high-impact discoveries over the past 50 years are scattered across many disciplines – including pediatrics, psychology, pediatric rehabilitation, epidemiology, and early childhood education. We thus have written this paper, designated as an “Hypothesis and Theory” contribution to Frontiers in Pediatrics, to present our vision for the practical application of these discoveries. We present an updated version of a conceptual theoretical framework that emphasizes a continuous learning system for optimizing the health and well-being of all young children through partnerships. This framework is compatible with a 2024 report from the JAMA Summit on Clinical Trials (1) that provides a clear vision with strategies for “modernizing the data infrastructure for clinical research to meet evolving demands for evidence” – recognizing the need to combine RCT results with real-world data documenting a far wider variety of clinical experiences and outcomes.

Major advances in maternal-fetal medicine, genetics/epigenetics, neonatology, pediatrics, developmental psychology, and functional neuroimaging have opened up new strategies to improve the monitoring and development of young children with neurodevelopmental risks and medical diagnoses. These advances are compatible with most biopsychosocial and ecological conceptual frameworks about human development, such as those proposed by Arnold Sameroff (2), Urie Bronfenbrenner (3, 4), the Rameys (5, 6), and Clancy Blair (7), among others. The World Health Organization International Classification of Functioning, Disability and Health (ICF) (8, 9) and the life course health models proposed by Halfon, Litt, Msall, Hirschfeld, and colleagues (10, 11) are additional examples of broad, integrative frameworks intended for use with clinical pediatric patient populations.

A critically important goal is increasing the awareness among medical and health care practitioners, as well as scientists and parents, of how useful these broad conceptual frameworks can be in making strategic reforms in the systems of care and partnership models of clinical decision-making related to improving child outcomes, including children's resiliency, risk reduction, functional abilities, learning, and neuroplasticity. Mark Scher (12), among others, has brought forth how the model of a dynamic multidimensional exposome – that is, the measure of all lifetime exposures of a child and how these relate to health at different times and cumulatively – plays a crucial role in lifespan brain health and, in turn, can transform the way we prepare clinicians for practice (13). In preparing this article, we have placed high value on published results from RCTs, yet we allow ourselves to share impressions from our direct experiences, including our community-based participatory research projects and working as lead clinical and scientific administrators within academic pediatric and rehabilitation service delivery systems in the United States. Above all, we recognize that widely accepted assumptions about children's development are strongly influenced by social pressures, professional traditions, and broad cultural factors, not only scientific evidence about child development and treatment efficacy. This reality often results in strong resistance to implementing scientific findings and new interventions that challenge or criticize the status quo. We nonetheless are optimistic that interdisciplinary partnerships and inclusion of people with lived experiences (14, 15) in our clinical service delivery and scientific investigations will facilitate more rapid, responsive, and successful implementation of evidence-based practices (EBPs). Although we particularly focus on children who face exceptional challenges, we conclude that evidence strongly favors the commonalities in how all children learn and in the supports that promote their optimal health and performance. That is, we endorse total-child practices and a shared conceptual framework for learning, functioning, and resiliency of all children, rather than developing entirely separate frameworks for specific medical diagnoses and environmental risks.

Our early intervention RCTs as well as our laboratory studies, have revealed and affirmed many basic neuroscientific principles for optimizing early childhood learning, health, and quality of life. These highlight the importance of dynamic, repeated, and responsive transactions that young children have with others and the environment – transactions in which young children actively participate and experience the immediate effects of their own behavior (responses) on what happens next. One of these central foundational principles of infant learning is known as “response-contingent learning” [e.g., (16, 17)] – a type of learning that has propelled the development of many efficacious interventions for vulnerable infants, including infants with failure-to-thrive (18), children born into extreme poverty with multiple environmental risks (6, 19), infants who are premature and low birthweight (20, 21), and children with cerebral palsy and other neuromotor impairments (22–25).

## Paradoxes in pediatric rehabilitation and early intervention programs

We have identified the following four paradoxes through our own longitudinal studies, clinical practice, and RCTs of young children with multiple risks and/or special needs. They also receive supportive evidence from many others [e.g., (26–32)]. We present each paradox in words conforming to the traditional definition as “a statement that is seemingly contradictory or opposed to common sense and yet is perhaps true” (33). Table 1 lists these along with their relationship to fundamental principles of development and their implications for hypotheses in treatment intervention and research.

**Table 1:** Major paradoxes from early intervention research and their treatment implications.

Paradoxes	Principles about development	Treatment implications
1. Recognizing the intertwined biological and environmental influences on a child's well-being leads to better functional outcomes than focusing primarily on medical diagnoses and biological risks. Conclusion: Biology and experience are transactional.	Conditions at birth, including genes, brain injury, and medical conditions, do not strongly predict long-term outcomes, unless the child's immediate and cumulative environmental conditions are considered. Prognoses about a child's future usually are based on prior clinical observation and reports. These can be limited and sometimes wrong, because many children in the past did not receive effective early treatment.	At birth, and sometimes prior to and during pregnancy, clinicians and parents should assess potential risks, the child's early learning opportunities, and specialized health supports. Measuring a child's progress should portray the child's overall health & functional outcomes, even by specialists focused on biomedical conditions. Treatment approaches plus child/family supports and risks should be shared promptly and fully with key individuals.
2. High-intensity, high-cost interventions that are well-timed, wholistic, and multi-domain can be less expensive (i.e., yield higher “returns on investment”) and better optimize life course outcomes than treatments that initially appear less costly, easier, and narrower. Conclusion: More-intensive treatments may cost less in the long run by improving multiple functional and health outcomes.	Children learn best when they initiate transactions that produce immediate, clear responses (response-contingent learning). High levels of varied practice and adult-guided shaping produce higher performance than lower levels. Treatment-induced neuroplasticity, including overcoming injury and adversity, can be stimulated by intensive interventions. Treatment's full impact cannot be adequately measured solely at end-of-treatment or by relying only on currently available general measures of child development.	Traditional delivery of pediatric rehabilitation and early education/early interventions in the U.S. will need to change to permit implementing proven effective high-intensity and multi-domain treatments. Many therapists and early educators will need specialized training in the rationale and specific techniques to implement many of the newer and proven treatments across health, home, education, and community settings. Insurance and clinic administrators need to be partners to help eliminate financial barriers to delivering EBPs.
3. Treatments can be individualized for a child while also adhering to systematic, evidence-backed treatment protocols. Conclusion: Individualized treatment and RCT protocols are compatible.	Treatment planning should engage the parents and child from the start, and consider their availability, treatment goals, communication preferences, & cultural/family priorities. Clinicians require clear guidelines to deliver EBPs with high fidelity to the tested protocols.	Implementing EBPs yields optimal results when therapists document the EBPs, the dose and treatment duration, and measures of progress toward goals. Treatment goals and how treatment is individualized should be recorded across health, rehabilitation and education systems.
4. A clearly presented conceptual framework can be remarkably practical, comprehensive, and powerful for improving outcomes, rather than merely an exercise in abstract theorizing. Conclusion: A shared conceptual framework can benefit families, therapists, health and educational practitioners, and scientists by providing a shared vision for their partnership.	Understanding needs of a child & family require clear and open sharing of assumptions about their current biobehavioral status, the family's supports & risks, and contextual influences of home, neighborhood and culture. Both visual and written forms of a conceptual framework can help with sharing data among key decisionmakers when planning and monitoring treatments. For research, a conceptual framework identifies which components of the child's and family's life are being targeted and measured at specified intervals.	A total-child framework (see text for definition) offers a flexible strategy for both the family and the health and education systems to view a child's progress over time (ideally, multi-year). Since children's transactions with others and the environment directly change their biobehavioral status, frequent and timely sharing of information as well as adjusting treatment are imperatives to achieve positive outcomes and supports for flourishing.

### Paradox 1: recognizing the intertwined biological and environmental influences on a child's well-being leads to better functional outcomes than focusing primarily on medical diagnoses and biological risks

These combined influences often are not simply additive but represent distinct and cumulative forces that optimize or impair life course health and developmental trajectories. Importantly, environmental supports influence how biology expresses itself during specific time periods of a child's life and how children's brains learn and adapt. This paradox challenges a conventional clinical assumption that there usually is one primary etiology (cause) that explains a child's impairment or diagnosed disability. Typically, this assumption of primary biomedical determination leads to a clinical diagnosis with a general prognosis about the child's future health and development. This diagnostic determination often is conducted by a physician who has little knowledge about the child's everyday environment, including the family home environment and parenting practices, and who seldom has spent enough time with the parents and child to understand how the child's “individuality” (such as comfort with strangers, willingness to try new and potentially difficult things, cooperation with adult or parental requests, ability to sustain focused attention to specific tasks) may influence the child's performance during standardized assessment sessions and clinical examination. In turn, after the physician completes the initial diagnostic workup, the child is referred to other specialists for further evaluations, largely so the child can receive treatments from rehabilitation specialists - such as physical, occupational, speech-language, and behavior therapists - and so a child's eligibility can be determined for special child care and early educational programs the family may choose to access. Remarkably, rarely is there a total-child assessment that adequately addresses this paradox of intertwined influences between biology and environment. Currently, many children receive a complex set of services, and may have a “multidisciplinary team” that gets together and agrees to a treatment-management plan. All too often, however, the resultant treatment plan is merely a combined set of separate interventions, not closely coordinated and not informed by a total-child perspective. Accordingly, opportunities for maximizing improvement in the child's biomedical condition and development across multiple domains are overlooked, fragmented, and seldom scaffolded to the child's joy of learning essential skills and task-mastery in everyday activities.

The emerging and enthusiastically embraced scientific developmental models referred to as “epigenetic models” seek to overcome the dual view of a child as an equation with separate elements for biology/genetics and the environment [cf (34) for overview]. Although debates about nature vs. nurture may continue in public arenas, discoveries of the past 50 years show that genes, for example, can be turned on, turned off, and influenced by experience in how they are expressed. For some people, this epigenetic framework becomes so technically complex that they may lament that “If everything affects everything, how can we ever make sense of a given child's life?” We have no simple answer nor do we deny it can feel overwhelming to consider a wide array of complex and simultaneous influences. Rather, we are encouraged by examples of complex biological and behavioral systems in the fields of cardiovascular health and cancer, for example, where specialists with different areas of expertise understand how important it is to include other simultaneous influences to maximize positive patient outcomes.

We have observed that when a young child has a diagnosed CNS disease, injury, or genetic aberration, the focus initially is on that condition and its management, rather than considering the child's health and functioning in other domains. This is often demonstrated in the management of children who were born extremely preterm. They experience a variety of complex medical conditions including bronchopulmonary dysplasia, necrotizing enterocolitis, high risk of sepsis, and feeding and regulatory behavior delays. Most importantly, the immature CNS of extreme prematurity has vulnerability to intraventricular hemorrhage, periventricular hemorrhagic infarction, periventricular leukomalacia, and retinopathy of prematurity. The neurodevelopmental outcomes of these children include elevated rates of cerebral palsy, neurosensory disability, global developmental delay, and challenges in coordination, visual perception, communication, executive function, and learning disorders [e.g., (10)]. Overall, outcomes are improving for very low birthweight and premature infants, and mounting evidence indicates the importance of environmental enrichment and family supports [e.g., (31)]. For instance, a cohort study of preterm infants (35) showed that maternal factors were as important as direct brain injury – resulting in the finding that among higher social status children there were *no* lasting effects of neonatal brain injury on measured cognitive outcomes.

Understanding the combined transactions among environmental supports from the child's family, community, and medical interventions is imperative. We seek to counter this situation by not focusing unduly on either the biological risks or environmental risks, because failure to see the “big picture” of a child's life *in its full context*, including cultural influences, can lead to selecting treatments that are not sufficiently informed about the natural supports and likely stressors impacting the child and family.

This paradox that biomedical conditions are inherently intertwined with the environment is affirmed in scores of studies in which pediatric interventions produce effects on aspects of the child's or family's life that were not explicitly targeted by the treatment. Often, when unpredicted positive changes occur in multiple domains, these are described as spillover effects, secondary outcomes, or pathway outcomes mediated by the treatment intervention. Many plausible explanations for these multi-domain effects exist. One explanation concerns how the brain develops, such that the “motor areas” of the brain can influence the “prefrontal or thinking areas” of the brain, or that the “emotional areas” of the brain can support or interfere with “decision-making areas” of the brain. Another focuses on the psychological view of the child as having voluntary control or “agency” over new behaviors: after a child observes making major gains in a short period of time, the child may try to master other difficult tasks that were not part of the original treatment. Or from a social ecological view, when parents and others in the child's life see rapid, large improvement in one area, they may increase their expectations about the child's future and begin offering new learning opportunities and supports for achievement that they previously had not considered. Perhaps one of the most defensible explanations of why a child improves in more than just one domain after receiving effective treatment is that during the intervention itself the adults engaged in new activities that promoted “incidental learning” and “observational learning.” One example from our work includes children with unilateral or asymmetric cerebral palsy who receive multiple weeks of full-day Constraint-Induced Movement Therapy (CIMT) concentrated on improving skillful use of the more impaired (paretic) arm-and-hand who then begin to walk, speak, initiate more social interactions, and/or show marked reduction in behavior problems (24). Another example of cross-domain benefits is from an RCT, known as the Abecedarian Project, with infants born into very low-resource, high-risk families who received 5 years of full-day, high-quality child care with an individually-paced educational curriculum, Learningames, informed by response-contingent principles. The children who received the Abecedarian Approach intervention showed not only significantly higher cognitive and language development in the early years (the primary targeted outcomes), followed by higher academic achievement in reading and math throughout the school years, but in middle age they showed greater compassion and caring for others in their decision-making activities, had more positive adult relationships with their parents, had significant differences in their brain structure, and had higher rates of full-time employment, advanced education attainment, and accumulation of material assets (6). Finally, another example is from cryosurgery for retinopathy of prematurity RCT, where successful ophthalmological interventions not only increased favorable visual status but also were associated with higher levels of motor, self-care, continency, and social cognitive functional skills (36). Importantly, favorable visual status and more optimal functioning at kindergarten entry predicted higher academic performance in reading, mathematics, and handwriting and a decreased need for special education placements at age 8 years (35).

One of the earliest examples of the dynamic play between biology and the environment is the Sameroff and Chandler (1975) (37) landmark article that reported the finding that low birthweight/premature infants from different socioeconomic circumstances had major differences in their long-term outcomes. Specifically, being born prematurely and/or low birthweight for children from economically impoverished families exerted a stronger negative consequence, when compared to peers from similarly impoverished families who were full-term, normal birthweight, than did similar degrees of prematurity/low birthweight for children from higher resource families, who often showed negligible or no long-term effects of this biological condition at birth. This does not mean there were no consequences of the infant's early birth and/or inadequate circumstances, but rather that an enriched family environment served to help overcome the potential harm of biological birth risk conditions. This observational finding was later confirmed in an 8-site RCT known as the Infant Health and Development Project that tested a multi-pronged early educational intervention for 985 premature, low birthweight infants through the first 3 years of life (20, 21). Overall, the early intervention – almost the same as that in the Abecedarian Project - demonstrated efficacy across all 8 sites. However, a more refined look showed sub-group differences: specifically, premature, low birthweight infants born to parents with at least a 4-year college degree performed equally well (above national average) whether or not they received the early education intervention. For all other maternal education groups, the intervention produced significant gains in cognitive scores at age 3 compared to the control group. We interpret this finding as supportive of the inference that high-resource families, even when in the control group, likely sought out and provided stimulation and care that effectively counteracted the potential long-term harm of prematurity and low birthweight.

#### Paradox 1 implications for practice and hypotheses

For children with biomedical conditions that are potentially complicated by environmental conditions, it is vital to consider how these multiple factors influence the accuracy and the impact of diagnoses, treatment recommendations, and child outcomes. To realize maximal benefits of treatments, adopting a whole-child perspective is likely to improve the feasibility and benefits of the child's overall treatment plan.

### Paradox 2: investing in high-intensity, high-cost interventions that are well-timed, wholistic, and multi-domain can be less expensive (i.e., yield higher “returns on investment”) and optimize life course outcomes better in the long-term than many treatments that initially appear less costly, easier, and narrower

What may at first appear to be a treatment that is too demanding – a high dose of therapy in a concentrated period of time - surprisingly can be easier and “more enjoyable” for the child, the family, and even the therapists or teachers than conventional therapy that is low dose and initially may appear simpler to deliver and less burdensome. An endorsement of this paradox is provided by independent teams that have conducted long-term or lifespan economic analyses adopting a “return on investment” (ROI) paradigm. For example, James Heckman and colleagues (38) completed an expansive set of ROI analyses, using longitudinal data over 4 decades from 2 independent RCTs we conducted - The Abecedarian Project and Project CARE. These studies tested the same multi-modal early intervention from birth to age 5, using the Learningames curriculum as part of the high-intensity intervention (full day educational child care, 5 days/week for 50 weeks/year for 5 years) for highly vulnerable children born into extreme poverty with multiple family-level risks. They concluded that each dollar invested in the Abecedarian Approach yielded an ROI of $7.30, producing an average annualized rate of return of 13.7 percent. Despite the relatively high initial cost (about the same or less than the cost of center-based Early Head Start programs and high-quality private childcare in the U.S.), this intervention prevented many non-optimal developmental and health outcomes that would have been far costlier *when placed into a life course framework.* In addition, we point out that the overall costs for high quality, comprehensive, intensive interventions are far less than for a 2 to 4-week inpatient rehabilitation stay for adults with stroke, traumatic brain, or spinal cord injury or a hip replacement and rehabilitation after a fall.

To date, this model of appraising the overall worth of high-intensity early interventions has not been applied systematically in pediatric rehabilitation. However, we strongly encourage this, including consideration of places where EBPs have been successfully adapted in spite of the early intervention and rehabilitation resources being limited and notable differences in the culture and community context for parenting [e.g., (32, 39)]. In the U.S., many children with neuromotor and/or cognitive disabilities receive nearly two decades of relatively low dose (a few hours per week), but mostly unproven forms of individual rehabilitation, amounting to thousands of hours of insurance or government paid treatment. In contrast, even repeated epochs of high-intensity rehabilitation that yield clinically significant benefits would be far less costly - in terms of both monetary and time costs - in the long run.

In a 2013 major comprehensive review of treatments for children with cerebral palsy, Novak and colleagues (27) opened with this provocative statement: “Thirty to 40% of interventions have no reported evidence base and, alarmingly, another 20% of interventions are ineffectual, unnecessary, or harmful (p. 885.)” By 2020, when they published a major update of findings from newly published studies (28), they were able to add many other treatments to the “green light” or “do it” interventions, although the majority (66%) were still in the “yellow light” or “apply with caution” category. We cite these two reviews because they are exemplary in seeking to synthesize results from the rapid increase in rigorous studies of the efficacy of a wide range of treatments. We also provide a quote from the end of their 2020 review, because it reflects the slow rate of implementation of new discoveries and continued reliance on traditional “ineffective” approaches: “There is a lack of robust clinical efficacy evidence for a large proportion of the interventions in use within standard care for people with cerebral palsy (p. 13)” (28). When considering cost implications of high dosage “green light” efficacious interventions, such as Bimanual Therapy and CIMT, we urge families, clinicians, and administrators to consider the large costs that already accumulate by delivering “standard care of uncertain benefit”.

We often have wondered if the improved functional and health outcomes for children with developmental disabilities after they receive high-intensity early interventions can be attributed to driving up their engagement in exploration, play, and participation throughout childhood? For example, children with Down syndrome once were expected to die at very young ages, and now they live into their 60s, clearly the result of both many medical advances and a completely different view of the potential of these children to learn and fully participate in a wide range of activities from childhood through adulthood (40).

### Paradox 3: treatments can be individualized for a child while also adhering to systematic, evidence-based treatment protocols

Our knowledge about efficacious behaviorally-based interventions for biologically and socially vulnerable young children are that the intervention protocols almost always specify that the treatment be adjusted for the child, via individualized treatment goals, pacing the treatment components to match the child's progress and interests, and altering the transactions to ensure the child and family stay highly engaged. These interventions emphasize the importance of children having fun, often embedding the structured learning and therapy activities into everyday play and typical routines, such as meals, dressing, and hygiene. Stated in other words, the majority of efficacious early interventions include explicit instructions for individualizing the treatment to the child, identifying what is rewarding and enjoyable for the child, and frequently monitoring the child's responses and progress during the course of treatment so that adjustments can be made as needed. In addition, many effective interventions consider the child's environment and other life circumstances in deciding when and where to provide treatment and how to effectively engage the child's family and other caregivers.

An important caveat to consider is that most journals strictly limit the number of words allowed in reporting results of clinical trials, resulting in extreme brevity when describing the treatment intervention. For example, the Infant Health and Development Program, an 8-site RCT for low birthweight, premature infants, involved a weekly home visiting program for the first 12 months of life (age-adjusted for prematurity), with a reduced frequency of home visits until age 3 years, and then from 12 to 36 months old providing center-based treatment for 5 full-days per week, 50 weeks/year in a specially designed and staffed child care center, using the Partners for Learning curriculum. This multi-domain intervention was based on informed developmental psychology science about which types of activities promote early learning at what ages in child care and home learning environments. This RCT included weekly documentation of the intervention and each child's progress as well as centralized monitoring with feedback to the providers at each local site. Training for the teachers and teacher assistants was intensive and included explicitly designed activities with monitoring for implementation fidelity. Also, the intervention included structured parent meetings that provided informational content and instructions for parents for home carryover, while also listening and responding to parents. In the first published article about the results (20), in the esteemed Journal of the American Medical Association (JAMA), the entire 3-year treatment intervention was described in only four paragraphs with fewer than 300 words! (Two later books providing extensive information about the treatment protocol required more than 1,000 pages; and the intervention is formally stored in NIH archives of efficacious early childhood interventions (41, 42). For clinicians and early childhood educators, a brief description of the treatment is woefully inadequate, failing to specify the content and timing of the formal “learning games” that teachers presented to the child and how these were paced sequentially, allowing activities within each domain to be continuously individualized for each child based on documenting the child's progress daily and weekly.

There also is a long history of implementing a variety of treatments, often deemed efficacious in well-controlled trials, that are based on principles from operant conditioning and formal learning theory, including Applied Behavioral Analysis or ABA treatments. This has led to some clinicians and families to mistakenly believe that any “standardized” intervention would be overly regimented and lack individualization. More than 20 years ago, the National Research Council issued a report “Educating Children with Autism” (43) that strongly affirmed the value of ABA-informed treatments as well as the necessity of high-dose treatment (a minimum of 25 h per week, 12 months a year) and individualized services to yield measurable and enduring benefits. This report resulted in a major transformation of the practices and insurance coverage for this pediatric patient population, as well as identification of key areas for future scientific inquiry. Even today, the insights from this exemplary project are worthy of careful consideration, especially their relevance to other groups of children with special needs.

A key to replicating treatments with high fidelity in real-world settings includes instruction for all individuals involved in delivering treatment. Ideally, this includes having specified standards for knowledge and behavioral skills of clinicians, parents, and others who participate. For example, in an ongoing Phase 3 randomized clinical trial conducted in 15 sites, The I-ACQUIRE Study funded by NIH, therapists receive centralized training and formal certification over several weeks (some self-paced and at least 3 full days of in-person demonstration and practice), followed by a written exam and coded observation of them delivering the I-ACQUIRE treatment. Then throughout the trial, each therapist submits at least one hour of videotaped treatment per week for each child; this is promptly scored by Master Therapists in the I-ACQUIRE intervention protocol who provide feedback within the next week to ensure that the protocol implementation continues to adhere to the key required elements. Embedded throughout this process is the necessity of *individualizing the treatment for each child*, maintaining the child's attention and active participation, and ensuring that treatment activities are fun, play-like, and combined with other naturally occurring daily activities, such as eating and dressing. In 2025, Jackman and colleagues (44) proposed having a process-oriented tool to help clinicians and organizations monitor their application of evidence-based practice guidelines, a promising idea that is worthy of field-testing to estimate its value in improving community implementation of treatments deemed efficacious.

#### Paradox 3 implications for practice and hypotheses

There is a widespread, but unfounded belief that providing a specified treatment protocol ignores the individuality of the child and family; in turn, this incorrect belief leads many clinicians to approach each child as a unique case who will need a unique array of treatment strategies. Almost all efficacious treatments build-in benchmarks, goals, and plans to adjust for the individual child while also adhering to the specified protocol, such as behavioral shaping, changing the pace of treatment delivery, or altering the natural learning opportunities for the child to attain more advanced levels that integrate and maintain new skills in the child's natural environment.

### Paradox 4: a clearly written conceptual framework can be remarkably practical and powerful, rather than merely an exercise in abstract theorizing

In psychology, Kurt Lewin is often cited for his idea that “There is nothing as practical as a good theory” (45). Lewin founded what is known as “Action Research” (1946) (46) and we re-affirm his maxim here. For pediatric rehabilitation, the theoretical or conceptual framework ideally will align closely with biopsychosocial and ecological models of brain adaptation and learning (e.g., refs cited above) and be compatible with the emerging area referred to as “precision rehabilitation” (22, 47). The details that accompany a complex, multi-user conceptual framework may be viewed at different levels of magnification and specification, akin to using a Global Positioning System application (GPS) for driving in a large urban area that is adjusted for close proximity vs. far distance viewing, knowing that at each level there is important and different information for use at different times and different purposes. Just as GPS programs require continuous and sometimes major updates, a strong conceptual framework must be amenable to revisions and refinements to provide the best currently available data.

Presently, the best-known framework used by therapists who work with children with developmental disabilities is the World Health Organization (WHO) International Classification of Functioning, Disability and Health – designated the ICF Framework (8). The ICF frequently is taught during pre-service and continuing education for occupational therapists, physical therapists, developmental and behavioral pediatricians, and physiatrists and often is alluded to in presentations at professional meetings. Further, the ICF has been used to identify important variables to collect systematically to encourage comparisons across electronic health data systems (note: this has occurred primarily in countries that have universal health care and provide rehabilitation services to all eligible children). Increasingly, however, critiques of the ICF have appeared (48, 49) - perhaps something that inevitably occurs after experience using a new system. That is, to be enduring and successful, a useful conceptual framework should explicitly be described as dynamic and open to incorporating changes, adding new constructs, and eliminating others when they no longer are sufficiently accurate or helpful. We have admired the amount of thought and work from the interdisciplinary team that created the ICF system, especially broadening beyond what many refer to as “traditional medical model” perspectives. Unfortunately, in the settings where we have worked directly in launching new multidisciplinary clinics and treatment programs, and in the research centers we have helped to build, we seldom observe clinicians referring to the ICF framework in their decision-making about individual children's treatment plans or using it to stimulate development and testing of new treatment approaches. Further, many neonatologists, pediatric neurologists, orthopedists, primary care pediatricians, and family practice physicians – that is, those primarily in charge of making the primary diagnoses for children with special needs - do not know much about or do not choose to use the ICF framework. One of the major reasons for gaps in using the ICF is that accessible measures of child functioning in daily activities at home and school and the supports they need for participation have not been uniformly applied in minimal data sets, in contrast to use of widely available measures for adult patients. Some useful tools for rehabilitation have been reviewed by Msall and colleagues over infancy, preschool, middle childhood, and adolescent developmental epochs (50).

The case against diagnosis-specific conceptual frameworks to understand life course development: We think that there are serious limitations in using a conceptual framework designed for “atypically developing” children or children with diagnosed “disabilities,” whether for a broad group of children or for specific diagnoses. Such disease- or disability-specific conceptual frameworks are based on an unstated assumption that the exceptionality/ies or diagnosed conditions for an individual child will continue, and that the strategies to improve a child's health, development, and well-being differ from models of learning and development for all children (that is, those considered “typically developing”). Research findings about the environmental forces that contribute positively or negatively to a child's current health and documented functional status are highly similar across many clinical diagnoses, ages, and contexts. Although we recognize that a child with a visible difference may more likely encounter negative social and administrative barriers to optimal functioning, “typical” children often experience somewhat similar barriers, usually more than once, as they grow up. How much these environmental forces will impact a child – regardless of the child's specific biomedical conditions and diagnostic labels – will be determined by a combination of biological and environmental influences. Thus, we strongly favor a general systems framework [see for an introductory overview of key principles of a theoretical systems frameworks in the life sciences, we recommend (51)]; for a more technical and classical treatise on systems theory refer to Bertalanffy (52).

The systems framework is well-suited to adding information about particular disorders, injuries, and risk conditions; just as importantly, this readily allows for the fact that most infants with a specific medical diagnosis will later receive additional diagnoses (often described as “co-morbidities” or “secondary conditions”). Imagine a child with a clinically confirmed neonatal stroke in the first 28 days of life who by 8 months old displays a clinically significant hemiparesis (major asymmetry in voluntary control over the left and right sides of the body, qualifying the child for a diagnosis of hemiparetic or unilateral cerebral palsy) and also demonstrates social and communicative behaviors at 24 months compatible with a diagnosis of an Autism Spectrum Disorder (ASD); then, a few years later this child begins to have seizures that require medication to control and earns sub-average scores on standardized tests of cognitive development and adaptive behavior that lead to another diagnosis - intellectual disability or global developmental delay. Furthermore, in school, the child struggles with attention and executive function and meets criteria for combined attention deficit hyperactivity disorder (ADHD). If we relied on separate diagnosis-specific conceptual frameworks for each of these conditions – that is, pediatric stroke, cerebral palsy, autism/ASD, seizure disorder, intellectual disability. and ADHD, this situation would be nearly overwhelming for the parents, the specialty therapists, the child's regular caregivers, early childhood teachers, and the child's primary care health practitioner(s). This hypothetical child example is far from rare or unfamiliar to those working with vulnerable young children. The complex neurodevelopmental disorders cannot be conceptualized in the same way as a focal brain injury in a mature brain, but rather these reflect a dynamic developmental brain that is simultaneously vulnerable and adaptable; accordingly, these likely will impact a spectrum of neurodevelopmental impairments in functioning. We also know that many children are referred for evaluation and then concurrently receive speech-language therapy, physical therapy, occupational therapy, high-intensity ABA therapy, and socialization/play therapy, each therapy at least once a week and sometimes multiple times per week. Perhaps even more importantly, we know that many children receiving multiple therapies from different specialists will later have one or more of their earlier diagnosed conditions “dropped” after they receive treatment(s) and as they become older. Over time, many of these children will encounter an array of changing social and environmental opportunities, as well as obstacles and stressors, that may exert an even more powerful effect on their quality of life and participation in meaningful, valued activities than did their earlier biomedical diagnoses. For such children and their families, we postulate that having a practically useful, comprehensive conceptual framework can help guide them over the years and across the changing health care systems and natural social ecologies.

The framework could be immensely helpful in (1) understanding their own child's development, (2) communicating with professionals from different disciplines about options for promising interventions and future preventive strategies that optimize functioning and participation, and (3) providing a life course perspective and appropriate accurate documentation as they navigate what is likely to be a complex set of decisions, treatments, and changing life circumstances. To the extent that true “individualization” is both a necessity and an ideal for all children, adopting a conceptual framework with plain language descriptions relating to a child's dynamic health and emerging behavior, skills, and motivation is decidedly not an “abstract” or “Ivory Tower” academic exercise.

In this Hypothesis and Theory paper, we present below a revised version of a conceptual framework that the Rameys and their colleagues have used in the design and conduct of >15 clinical trials in developmental disabilities and early childhood education (6, 53). This is based on a biosocial-ecological systems framework that has been instrumental in the design and conduct of multi-pronged and often high-dose interventions that we have tested with vulnerable infants, including failure-to-thrive infants, premature and low birthweight infants, and infants born full-term and healthy but who face multiple serious family and environmental risks to their well-being. We do not consider this to be a final framework, but rather offer this as a foundational platform designed to identify a variety of ways to promote mechanisms that can positively influence the child and family, in the short- and long-term.

#### Paradox 4 implications for practice and hypotheses

The agreement to use a written and illustrated conceptual framework as an organizing system for major planning and treatment choices, as well as for research, also is a commitment by participants to collect and review data about key indicators of child progress and to use this information to modify treatment – spanning biomedical and social environmental interventions – based on reliable, valid information. This necessitates that the family and lead clinical players discuss the framework and what it means to them. The conceptual framework serves as a unifying reminder of the importance of embracing a “total-child” perspective and committing to a longitudinal and life course view.

The paradox that a complex, multi-component, and transdisciplinary conceptual framework can be practical does not minimize the need for investing considerable time and effort to learn about the framework and develop both procedures and consensus practical outcomes they will use for monitoring over time and across situations. We have seen benefits from consistently using appropriate measures of child motor, cognitive, communicative, self-care, and social emotional functioning, as well as general health and well-being. We recommend that those who engage in this endeavor consider the recommendations that have emerged from reviewing “the science of team science” (54) that emphasize the critical importance of establishing a common shared language and learning about the value of highly specialized knowledge being integrated into a much broader or holistic perspective when seeking to advance understanding of a complex topic.

### The IMPACT2 framework: description of a flexible biosocial and ecological framework for promoting optimal development for children and their families

**Figure 1:**
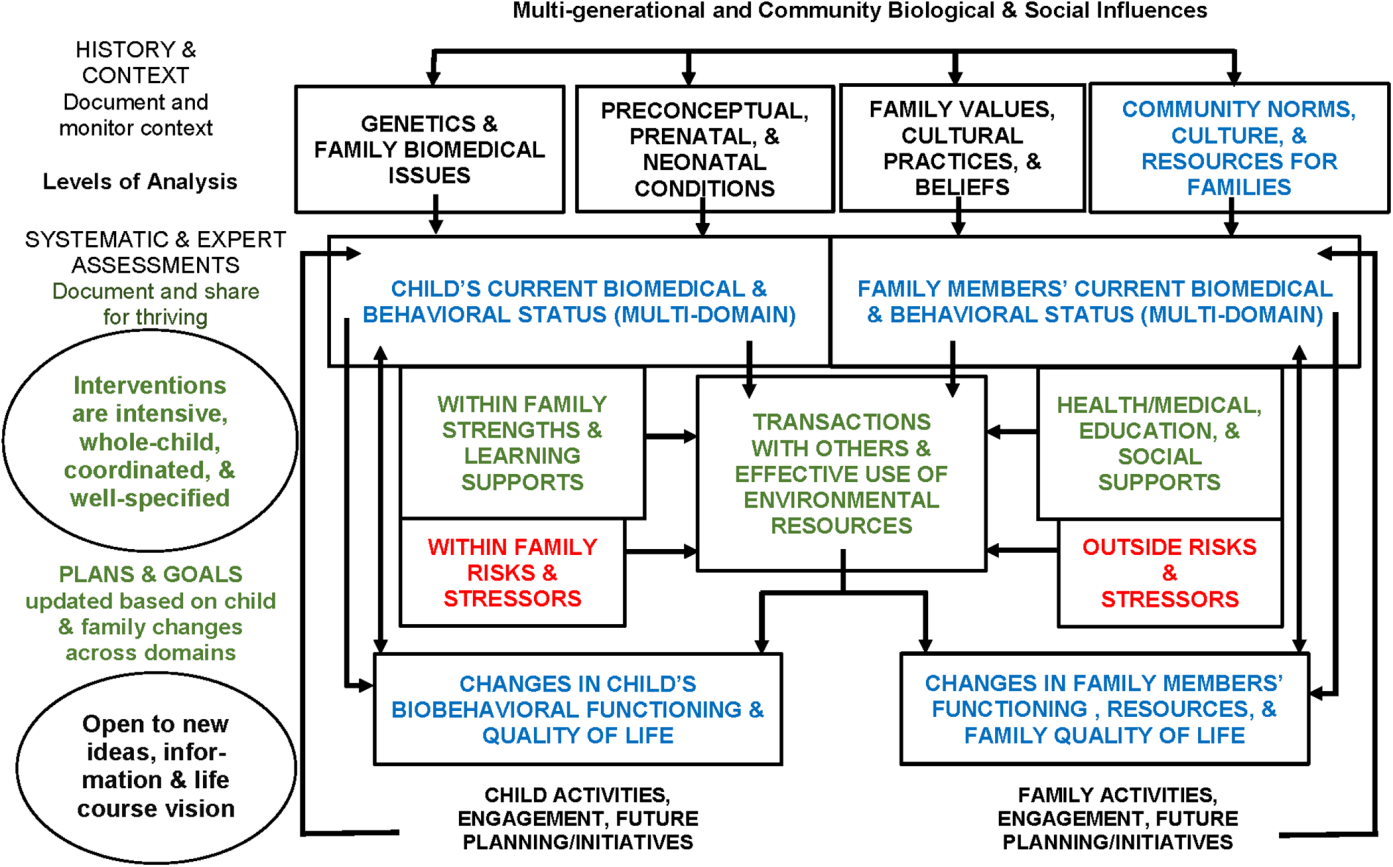
The IMPACT2 Lifespan Framework [informed by Biosocial Developmental Contextualism, C. Ramey & S. Ramey, 1998 (5)]. Key: Green = protect and promote; Red = prevent and reduce; Blue = monitor for changes and planning.

IMPACT2 is a total-child conceptual framework that builds upon earlier versions of the biosocial contextual development system that Craig Ramey and many colleagues have used since the early 1970s in the design and conduct of many RCTs [e.g., (6, 53)]. Here we choose the name IMPACT2 for this framework, to represent our intention that it is designed to facilitate a positive “impact” on the child and family. We also use IMPACT2 as an acronym capturing its endorsement of “Interdisciplinary Monitoring, Planning, and Caring for the Total-Child-Together.” One of the most distinctive features of IMPACT2 is that it identifies “context” as an active component, not merely a background or static description of the where and what of a child's and family's life situation. That is, context refers to a dynamic and potentially modifiable component – spanning many dimensions of the environment and culture - to consider in the design and measurement of new treatments. This framework is one that intentionally was designed to transcend any given culture, country, or service delivery models, and has been helpful in developing and testing locally-adapted variations of the Abecedarian Approach in 8 quite diverse countries (32) and helpful as a backdrop for our working in developing countries that have reached out to receive assistance in learning about EBPs so they can make cultural adaptations and best utilize their local clinical and community resources [e.g., (39)] At the heart of IMPACT2 is its emphasis on the child's “transactions with others and effective use of environmental supports.” Figure 1 illustrates the major constructs or components of the system.

#### Definitions related to using IMPACT2

*First*, we use the term “total-child” to designate that a child's well-being must simultaneously consider multiple domains of the child's life, including health, neuromotor, cognitive, language, social-emotional, and self-help domains, along with personal perceptions of well-being and quality of life. Essentially, the terms whole child, holistic child approach, and entire child connote similar meaning. S*econd*, the term “development” refers to changes that can be measured within specific domains and that promote maturation, flexibility, and participation in age-appropriate activities. *Third,* the construct of “transactions” derives from developmental science, encompassing broad sets of behaviors in which the child has an active role that potentially alters the other person(s) and/or the environment, and vice versa. Transactions are multi-directional influences that function as powerful mechanisms for children to learn about their role in co-determining their experiences and, in turn, their developmental outcomes. *Fourth,* adopting a total-child view means that skills and activities are not practiced solely under the auspices of one discipline or one setting. Examples: The child must move at home, in rehab, in group care, and school and community settings; parents and other adults must understand and promote the child's communicative and social interactive skills, not just speech therapists or psychologists; and physical health depends on many family-based choices and behaviors as well as what physicians, other health care practitioners, and teachers do for children. *Fifth and finally*, the word “together” at the end of the IMPACT2 name is essential to our proposed framework; optimizing a child's life course requires an active partnership involving parents, rehabilitation therapists, health professionals, educators, and community individuals (e.g., relatives, sports coaches, music and art teachers).

#### A beginning guide to looking at IMPACT2

We think of IMPACT2 as having features similar to a GPS that helps guide users from one place to another. The starting place is illustrated in Figure 1 in the box “Child's current biomedical and behavioral status, including risks (multi-domain,” while the designated destination is the box shown as “Changes in child's biobehavioral status and quality of life.” Figure 1 shows the large view or map, but any feature in the framework can be honed in on to identify greater detail about that place or construct, including measurement options. Most GPS systems allow the user to indicate preferences that help to select the final guidance directions (e.g., favoring shortest time vs. shortest distance, avoiding toll roads, adding in scenic routes/cultural attractions). Above all, most GPS systems have the option for users to keep track of prior destinations. Keeping track of prior journeys and their outcomes is what families do for all of their children as they grow up. Observing and continuously updating their interpretation of their child's transactions with associated consequences are ways that families, other key adults, and service providers make choices related to the child. Ideally, a practically useful interdisciplinary conceptual framework would efficiently store data about the child's and the family's relevant history, and facilitate identifying “routes” that did or did not work well for the users, and why (if possible). The experience of using these routes would include understanding of what was easy to implement, what tasks needed specific scaffolding, and what areas required pragmatic enriched environments (adaptive playgrounds, communication boards, alternative mobility). To date, however, no such system exists, although the technology is widely available and increasingly considered user-friendly.

We reminisce about the huge promise several decades ago about the revolution that would accompany individualized electronic health care records, while noting that navigating across service providers still has not become streamlined or easy for families whose child has a complex and shifting clinical profile. All too often, the information collected still remains in silos, rarely is fully shared or trusted across providers, and often contains serious errors that are difficult for parents or outside experts to correct. In the United States, this promise has not been realized largely because most families deal with multiple provider-specific electronic information systems, and other relevant publicly maintained databases (such as schools, public welfare services, and social supports) are not accessible directly by families or health providers. We have listened to parents and clinicians alike who lament this situation, still hoping that this promise will be fulfilled. Ideally, when partnerships commit to open data-sharing and including parents as full partners in the electronic archiving of essential information, then timely sharing of crucial data will occur to facilitate decision-making for individual children and families. These individual clinical records will also complement results from RCTs so that collectively we will advance understanding different developmental pathways for patient groups with shared characteristics and special needs, as recommended by the recent Summit on Clinical Trials (1).

We realize that this paper alone cannot serve as an adequate administrative guide for all the ways that IMPACT2 might be able to improve services and outcomes for a child and family. Rather, we report that this framework has directly helped us in designing multiple new interventions and in measuring their impact more widely and longitudinally. From the start, this framework emphasized the potentially powerful role of context and culture, the inseparable nature of biological and behavioral development, and the importance of frequent measurement of what is happening in a child's life, including both formal treatments and everyday environmental influences. As Figure 1 shows in the oval at the bottom of the far-left column labeled “Level of Analysis,” we know firsthand the rewards of working with interdisciplinary teams of practitioners, families, and scientists who are truly “open to new information, ideas, and a life course view.”

## Discussion

We identified four major paradoxes related to the science of early child development and the practice of pediatric rehabilitation. Our goal was to increase awareness of these paradoxes and how they truly change the dominant and traditional approaches for optimizing outcomes for children with special needs. We need to explicitly recognize and share with parents how strongly a child's biomedical status depends on external environmental influences (either improving or worsening the long-term consequences of physical and genetic differences) (55), rather than continuing to focus narrowly on just the child's medical diagnoses and risk conditions (Paradox 1). We also must recognize that many of the most impactful early interventions have been far more intensive (such as high dose and longer duration) and extensive (touching multiple domains of development, not just one or two areas) (55) (Paradox 2). Many clinicians continue to reject implementing these high-intensity treatment approaches using the excuse that they are too expensive (at least in the short-run) and require changing the way that traditional rehabilitation is delivered (in the U.S., this is often in clinics, during one-hour sessions, usually once or twice a week, and seldom directly coordinated with other activities in the child's life), then we predict that the status quo, described so aptly by Novak et al. in 2013 (27, 28), will directly limit the lifelong health and well-being of many children with special needs. A recent comparative efficacy RCT of dosage of pediatric CIMT, for instance, showed that even receiving three 2.5 h sessions of CIMT a week for 4 weeks (a weekly dose of 7.5 h) was *not* significantly better than Usual and Customary Treatment, but receiving five 3 h sessions (i.e., daily) per week for 4 weeks (a weekly dose of 15 h) did produce significant gains lasting at least 6-months for children with unilateral or hemiparetic cerebral palsy (25).

Another excuse we hear often from practitioners and academic directors of training programs is that their specialized type of rehabilitation requires “individualization” for each child and therefore they cannot be expected to deliver a “standardized treatment protocol.” This is incorrect (Paradox 3)! Finally, despite tremendous awareness that a child's outcomes are determined by a multitude of factors, clinicians rarely commit to an interdisciplinary partnership that openly discusses their different approaches and shows a willingness to speak up and change how they fulfill their distinctive roles. We propose that a shared conceptual framework is urgently needed (Paradox 4). Yet we are not naïve about the tremendous effort required to have active partnerships realized and maintained. The excuses for not being able to sustain these partnerships are abundant, as well as predictable. Further, many clinicians and parents feel that the “mandated” teams in the U.S. for developing treatment plans in early intervention and special education have been mostly a failure, and very costly. Our optimism propels us to persevere in seeking to have these partnerships work better than before.

**Figure 2:**
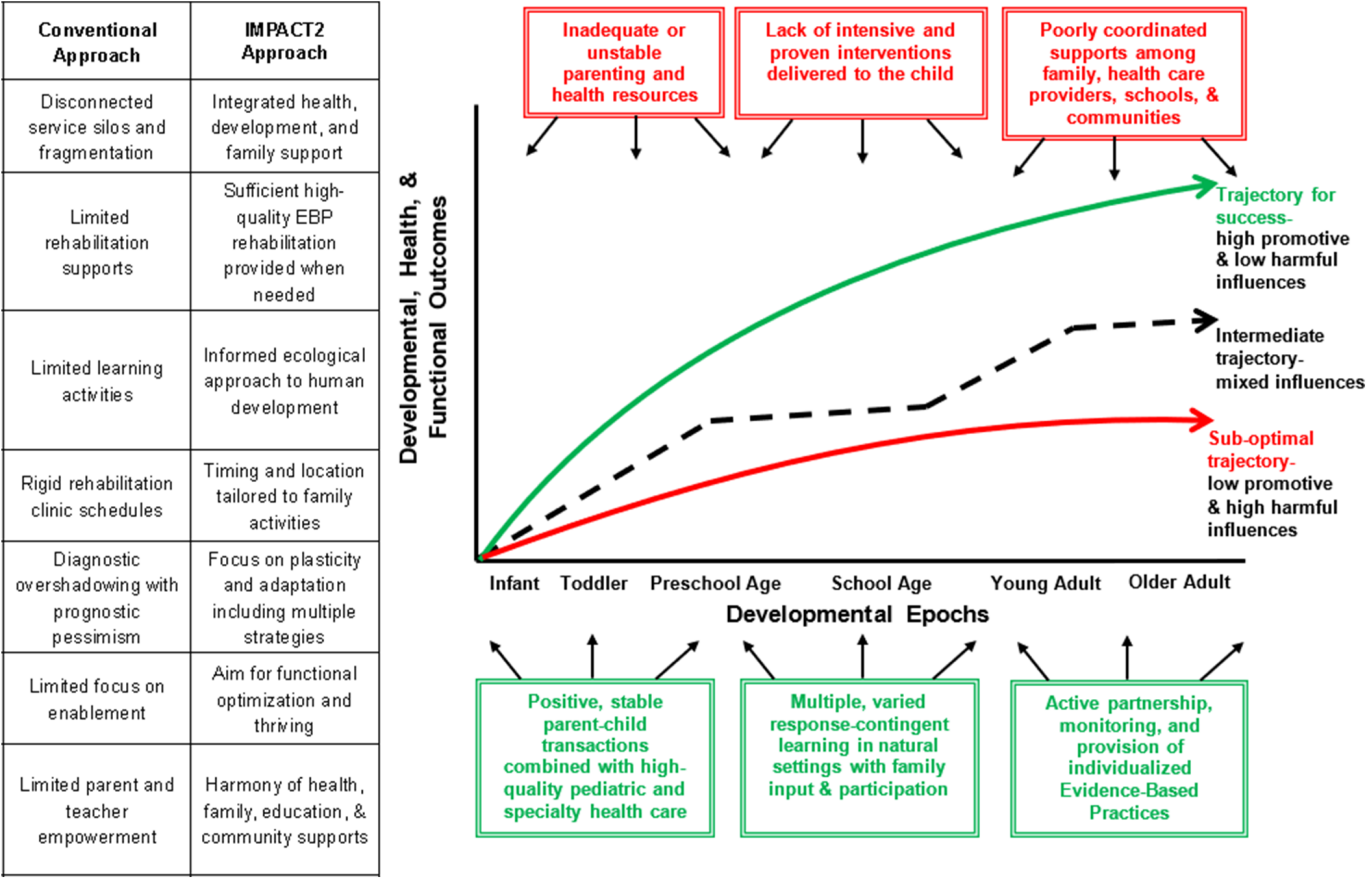
IMPACT2 Neuroplasticity trajectories: optimizing treatment outcomes, health, and quality of life for prematurity, cerebral palsy, Down syndrome, and other developmental delays and disablities (Key: examples of promotive influences in green boxes, harmful influences in red boxes).

We think the breakthroughs in understanding treatment-induced neuroplasticity and discovering efficacious forms of pediatric rehabilitation are important. We actively advocate for conducting more vigorous research and testing of novel and combination forms of therapy and enhancing parent and community supports – through interdisciplinary teams that include people with lived experiences. The days of giving parents dismal prognoses for a child's diagnosis, based on little and often outdated data, need to end. The strides seen in the life course outcomes for many children with diagnosed disabilities and serious biological conditions need to be celebrated and, most importantly, replicated. Perhaps it is time that we also cease to describe any medical condition as “static” and become receptive to changing the service delivery system in ways that listen to and act upon what parents and children have to say, what the findings of careful studies demonstrate, and what practitioners report has and has not worked for them. In closing, we share Figure 2 as an illustration that depicts the wide range of different outcomes possible based on the presence (or absence) of multiple co-existing factors in a child's life. We welcome comments and dialogue with readers!

## Data Availability

The original contributions presented in the study are included in the article/Supplementary Material, further inquiries can be directed to the corresponding author.
